# Three new species of *Pseudorhabdosynochus* (Monogenea, Diplectanidae) from several species of *Cephalopholis* and *Epinephelus* (Perciformes, Serranidae) from Thailand

**DOI:** 10.1051/parasite/2022049

**Published:** 2022-10-25

**Authors:** Chompunooch Saengpheng, Watchariya Purivirojkul

**Affiliations:** 1 Animal Systematics and Ecology Speciality Research Unit (ASESRU), Department of Zoology, Faculty of Science, Kasetsart University 50 Ngam Wong Wan Road, Chatuchak Bangkok 10900 Thailand; 2 Biodiversity Center Kasetsart University (BDCKU) Bangkok 10900 Thailand

**Keywords:** *Pseudorhabdosynochus suratthaniensis*, *Pseudorhabdosynochus cephalopholi*, *Pseudorhabdosynochus samaesarnensis*, *Cephalopholis argus*, *Cephalopholis sonnerati*, *Epinephelus lanceolatus*

## Abstract

*Pseudorhabdosynochus suratthaniensis* n. sp. is described from the gills of *Cephalopholis argus*; *P. cephalopholi* n. sp., from the gills of *C. sonnerati*; and *P. samaesarnensis* n. sp., from the gills of *Epinephelus lanceolatus*. These fish were all caught in the Gulf of Thailand. *Pseudorhabdosynochus suratthaniensis* n. sp. is distinguished from congeneric species by the structure of its sclerotized vagina, which has a wide sclerotized trumpet and a single large primary chamber, and by the number of rows of rodlets in each of its squamodiscs. *Pseudorhabdosynochus cephalopholi* n. sp. is also distinguished by the structure of its sclerotized vagina that, like the *P. suratthaniensis* n. sp., has a sclerotized trumpet, but it also has a long coiled or curved primary canal near its midlength, and a distal part with a primary chamber and a secondary chamber communicating with the primary chamber through a short secondary canal. In addition, *P. cephalopholi* n. sp. is distinguished by some sclerotized organs (ventral and dorsal hamuli, ventral bar, and quadriloculate organ) with different lengths, and by the number of rows of rodlets in each of its squamodiscs. *Pseudorhabdosynochus samaesarnensis* n. sp. is distinguished by its sclerotized vagina that has an anterior cup-shaped trumpet and a short straight or curved primary canal. For Thailand, these are the first species of *Pseudorhabdosynochus* described from species of *Cephalopholis* and the second species of *Pseudorhabdosynochus* described from *Epinephelus*.

## Abstract (fr)

*Pseudorhabdosynochus suratthaniensis* n. sp. est décrit à partir des branchies de *Cephalopholis argus*, *P. cephalopholi* n. sp. des branchies de *C. sonnerati*, et *P. samaesarnensis* n. sp. des branchies d’*Epinephelus lanceolatus*. Ces poissons ont tous été pêchés dans le golfe de Thaïlande. *Pseudorhabdosynochus suratthaniensis* n. sp. se distingue des espèces congénères par la structure de son vagin sclérifié, qui possède une large trompette sclérifiée et une seule grande chambre primaire, et par le nombre des rangées de bâtonnets dans chacun de ses squamodisques. *Pseudorhabdosynochus cephalopholi* n. sp. se distingue également par la structure de son vagin sclérifié qui, comme *P. suratthaniensis* n. sp., a une trompette sclérifiée, mais a également un long canal primaire enroulé ou incurvé près de sa mi-longueur et une partie distale avec une chambre primaire et une chambre secondaire communiquant avec la chambre primaire par un court canal secondaire. De plus, *P. cephalopholi* n. sp. se distingue par certains organes sclérifiés (hamuli ventraux et dorsaux, barre ventrale et organe quadriloculé) de longueurs différentes, et par le nombre des rangées de bâtonnets dans chacun de ses squamodisques. *Pseudorhabdosynochus samaesarnensis* n. sp. se distingue par son vagin sclérifié qui a une trompette antérieure en forme de coupe et un court canal primaire droit ou courbe. Pour la Thaïlande, il s’agit de la première espèce de *Pseudorhabdosynochus* décrite à partir d’espèces de *Cephalopholis* et de la deuxième espèce de *Pseudorhabdosynochus* décrite à partir d’*Epinephelus*.

## Introduction

Groupers (Serranidae, Epinephelinae) are bottom-associated fishes found in tropical and subtropical seas. Most species occur in coral reefs, but some live in estuaries or on rocky reefs [11]. The Epinephelinae are divided into five tribes that comprise about 234 species of marine fishes in 32 genera [28]. The hybrid grouper, a cross of the female tiger grouper *E. fuscoguttatus* and the male giant grouper *E. lanceolatus* (tiger grouper × giant grouper or TGGG), has taken the Asian aquaculture industry by storm since 2006. TGGG is widely cited as the most successful hybrid combination, as it is able to grow quickly [2, 6]. In Thailand, the peacock hind *Cephalopholis argus* (Bloch & Schneider), the tomato hind *C. sonnerati* (Valenciennes), the coral hind *C. miniata* (Forsskål), the giant grouper *Epinephelus lanceolatus* (Bloch), and the whitespotted grouper *E. coeruleopunctatus* (Bloch) occur in coral reefs and other coastal and offshore areas; the malabar grouper *E. malabaricus* (Bloch & Schneider) occurs in mangroves; the cloudy grouper *E. erythrurus* (Valenciennes) occurs in seagrass beds; and the orange-spotted grouper *E. coioides* (Hamilton) occurs in both mangroves and seagrass beds [29]. Species of *Pseudorhabdosynochus* Yamaguti, 1958 are mostly found on the gills of groupers mainly of the genus *Epinephelus* and appear to be specific to their host [18, 22]. *Pseudorhabdosynochus* currently has 96 valid species [10]. Four of those species – *P. argus* Justine, 2007, *P. minutus* Justine, 2007, *P. urceolus* Mendoza-Franco, Violante-González & Herrera, 2011, and *P. meganmarieae* Kritsky, Bakenhaster & Adams, 2015 – have been described from four species of *Cephalopholis – C. argus*, *C. sonnerati*, *C. panamensis* (Steindachner), and *C. cruentata* (Lacepède) [15, 24, 26], respectively. In this paper, we describe three new species – *P. suratthaniensis* n. sp. found on the gills of *C*. *argus*, *P*. *cephalopholi* n. sp. found on the gills of *C. sonnerati*, and *P. samaesarnensis* n. sp. found on the gills of *E. lanceolatus –* in the Gulf of Thailand. *Pseudorhabdosynochus argus* and *P. minutus* had earlier been described from *C. argus* and *C. sonnerati* off Nouméa, New Caledonia [15], respectively; in this paper, we describe a second species of *Pseudorhabdosynochus* from these fish. In addition, we describe the first species of *Pseudorhabdosynochus* from *E. lanceolatus*: *Pseudorhabdosynochus samaesarnensis* n. sp.

### Materials and methods

One specimen of *C. argus* (total length, 303 mm and weight, 443 g) was obtained from a jetty in Surat Thani province, Southern Thailand in June 2019. Four specimens of *C. sonnerati* (total length, 230–247 mm and weight, 234–287 g), and nine specimens of *C. miniata* (total length, 210–340 mm and weight, 169–637 g) were obtained from a jetty in Surat Thani province, Southern Thailand in May 2019. One specimen of *E. lanceolatus* (total length, 520 mm and weight, 5000 g) was obtained from a local fisherman on Samaesarn Island in the Gulf of Thailand, Chonburi province, Eastern Thailand in September 2020. Four specimens of *E. coeruleopunctatus* (total length, 290–330 mm and weight, 378–557 g), four specimens of *E. coioides* (total length, 350–470 mm and weight, 617–1500 g), five specimens of *E. erythrurus* (total length, 245–275 mm and weight, 260–338 g), four specimens of *E. malabaricus* (total length, 360–370 mm and weight, 759–844 g), and eleven specimens of *E. fuscoguttatus* (total length, 250–350 mm and weight, 270–914 g) were obtained from a local fisherman on Libong Island in the Andaman Sea, Trang province, Southern Thailand in April 2020. Three sea cage-cultured hybrid groupers (TGGG) (*E. fuscoguttatus ♀ × E. lanceolatus* ♂) (total length, 380–480 mm and weight, 1100–2600 g) were obtained from a local farmer in Ban Laem Hin, Phang-Nga province in the Andaman Sea, Southern Thailand in May 2020. All the fish were dead and were immediately transported in a cool box to the laboratory. Their gills were removed and placed in Petri dishes that contained seawater. Monogeneans were individually picked off the gills with a fine needle with the aid of a stereomicroscope and put on slides. They were prepared with ammonium picrate-glycerin, referred to as “Picrate” (see [13]) according to [25]. Then, their soft internal organs and haptoral hard parts were immediately examined. The slides were later sealed with Canada balsam [13]. Specimens were photographed using an Olympus DP 70 microscope (Olympus Corporation, Japan) and a Zeiss Axiocam 506 color microscope (Carl Zeiss AG, Germany) for drawing. Various sclerotized organs were measured based on a previous study (see Fig. 1 in [13, 14]; their nomenclature follows [14]). Measurements are in μm and given as mean followed by (minimum–maximum, *n*=) between parentheses. Specimens were deposited in Lee Kong Chian Natural History Museum National University of Singapore (herein abbreviated as ZRC), Natural “”History Museum, National Science Museum, Technopolis, Pathum Thani Province, Thailand (herein abbreviated as THNHM) and Zoological Museum, Kasetsart University (herein abbreviated as ZMKU).

**Figure 1:**
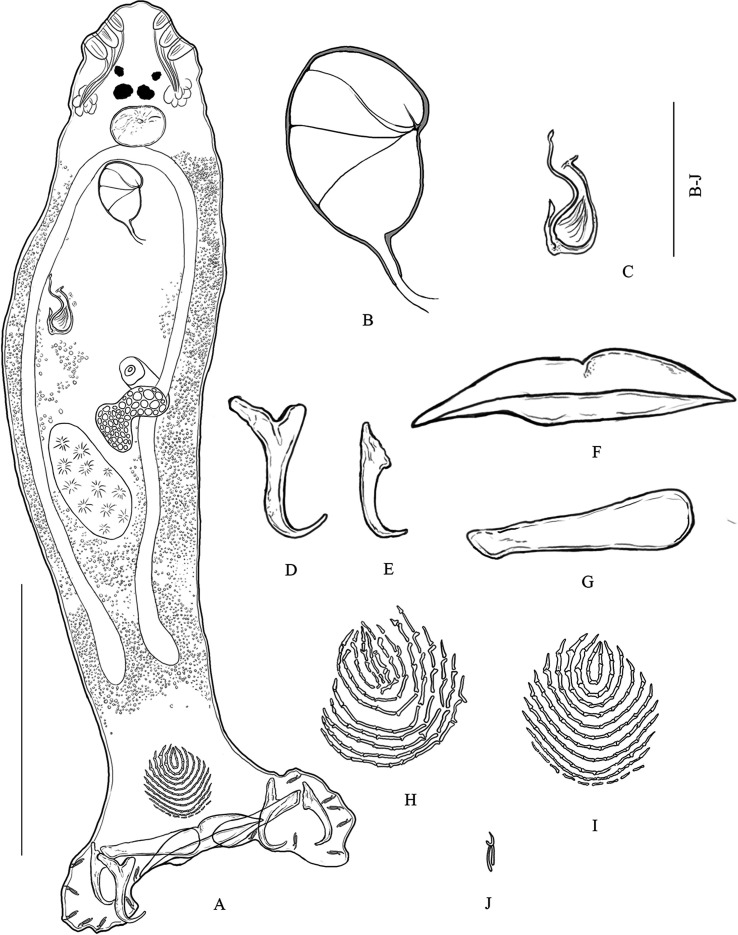
*Pseudorhabdosynochus suratthaniensis* n. sp. from *Cephalopholis argus* in the lower Gulf of Thailand. (A) COMPOSITE drawing (mainly from holotype), dorsal view. (B) Male quadriloculate organ, dorsal view. (C) Sclerotized vagina, dorsal view. (D) Ventral hamulus. (E) Dorsal hamulus. (F) Ventral bar. (G) Dorsal (lateral) bar. (H) Ventral squamodisc. (I) Dorsal squamodisc. (J) Hooklet. Scale-bars: (A) 200 μm; (B)–(J) 50 μm.

### *Pseudorhabdosynochus suratthaniensis* n. sp.

urn:lsid:zoobank.org:act:69811A09-7C5C-4D38-B2CA-72D77973425C

*Type-host*: *Cephalopholis argus* (Bloch & Schneider) (Perciformes, Serranidae).

*Type-locality*: Surat Thani Province, the lower Gulf of Thailand, Southern Thailand (9°49^′^10.1^″^N 99°55^′^31.1^″^E), June 2019.

*Type-material*: Holotype, ZRC.PLA.1115; 2 paratypes, ZRC.PLA.1116-17; 2 paratypes, THNHM-Iv-19363-64; 15 paratypes, ZMKU-PM-002039-53.

*Site in host*: Gills.

*Infection indices*: Prevalence 100% (one specimen examined and infected); 36 helminth specimens on the single grouper examined.

*Etymology*: The species name “*suratthaniensis*” is treated as an adjective and was derived from the name of the province “Surat Thani”, where the host fish *Cephalopholis argus* was collected.

#### Description (Figs. 1 and 2)

[Based on 20 specimens]. Body (including haptor) 759 long (619–977, *n* = 20); maximum width 169 (121–216, *n* = 20). Tegument smooth. Anterior region with 3 pairs of lateral head organs and 2 pairs of eye-spots; anterior pair smaller than posterior pair. Pharynx median, spherical, 45 (32–54, *n* = 20) × 45 (30–54, *n* = 20). Esophagus absent. Intestinal bifurcation immediately follows pharynx. Haptor differentiated from rest of body, 222 wide (177–277, *n* = 20), with 2 similar squamodiscs, 2 pairs of lateral hamuli, 3 bars, and 14 marginal hooklets. Dorsal and ventral squamodiscs round-shaped, made up of rows of rodlets, 1–2 central rows oval and closed. Dorsal squamodisc 53 long (41–64, *n* = 20), 55 wide (40–67, *n* = 20), with 9–11 rows of rodlets, of which the 1–2 innermost rows form closed ovals. Ventral squamodisc 59 long (47–73, *n* = 20), 57 wide (41–69, *n* = 20), with 8–11 rows of rodlets, of which the 1–2 innermost rows form closed ovals. Ventral hamulus with distinct guard and expanded deep root, elongated shaft slightly arched and recurved toward the tip, outer length 49 (44–53, *n* = 20), inner length 43 (39–47, *n* = 20). Dorsal hamulus with indistinct guard and expanded deep root, elongated straight shaft and recurved toward the tip, outer length 42 (40–46, *n* = 20), inner length 27 (25–31, *n* = 20). Dorsal (lateral) bar straight, with flattened medial extremity and cylindrical lateral extremity, 69 long (56–72, *n* = 20), 19 wide (16–22, *n* = 20). Ventral bar 93 long (85–100, *n* = 20), 18 wide (15–21, *n* = 20), with constricted median portion, pointed ends and visible groove that extends to both thin extremities. Male quadriloculate organ divided into 4 chambers, inner length 73 (65–84, *n* = 20), fourth chamber ends in sclerotized cone, 19 long (14–22, *n* = 20), prolonged by sclerotized tube, 19 long (16–23, *n* = 20), end of tube prolonged by filament of variable length. Testis subspherical, intercecal. Ovary pretesticular, encircles right intestinal cecum. Vitelline follicles lateral, coextensive with intestinal ceca and confluent in large zone posterior to testis and terminate anterior to peduncle, leaving free space around squamodiscs. Egg not seen.

**Figure 2:**
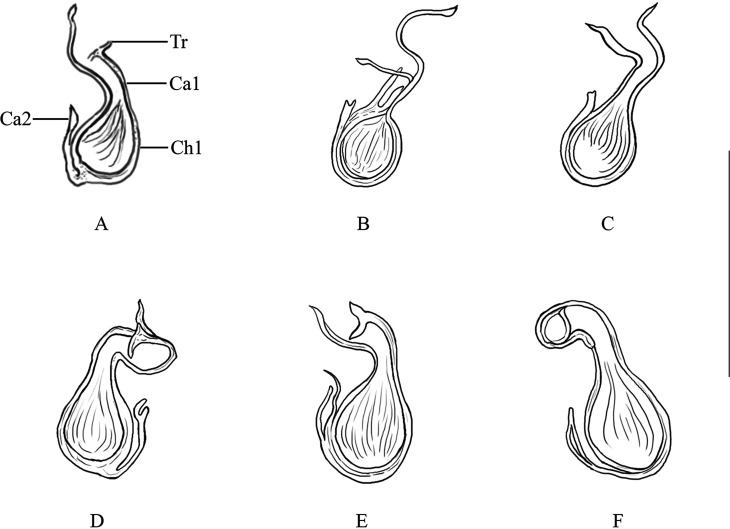
Variations (A)–(F) of the sclerotized vagina of *Pseudorhabdosynochus suratthaniensis* n. sp. from *Cephalopholis argus* in the lower Gulf of Thailand. Abbreviations: Tr – trumpet, Ca1 – primary canal, Ch1 – primary chamber, Ca2 – secondary canal. Scale-bar: 50 μm.

*Sclerotized vagina* with a complex structure, aspect changes slightly according to specimen and orientation (Figs. 2A–2F). Sclerotized vagina comprises anterior trumpet, followed by primary canal, primary chamber and secondary canal; trumpet in continuity with primary canal short, straight, or curved (Figs. 2A and 2D), heavily sclerotized and widens into single large and thick-walled primary chamber; secondary canal connected to primary chamber around base of vagina. Total length of sclerotized vagina (measured from distal extremity of trumpet to base of vagina) 40 (36–45, *n* = 20), length variable because of variation in curvature of primary canal. Primary chamber subspherical 19 long (15–23, *n* = 20), 17 wide (16–20, *n* = 20).

#### Differential diagnosis

*Pseudorhabdosynochus suratthaniensis* n. sp. is easily distinguished from other species of *Pseudorhabdosynochus* by the structure of its sclerotized vagina and the number of rows of rodlets in each of its squamodiscs. Another *Pseudorhabdosynochus* species that has a vaginal structure similar to that of *P. suratthaniensis* n. sp. is *P. urceolus* from *C. panamensis* from Taboga Island in Panama. The general structure of the sclerotized vagina appeared to be similar to that of the *P. suratthaniensis* n. sp. *Pseudorhabdosynochus urceolus* can be distinguished by the following characteristics: the size of its sclerotized vagina (29 versus 40 μm in *P. suratthaniensis* n. sp.); the morphology of its sclerotized vagina, with a bell-shaped opening that is not in *P. suratthaniensis* n. sp; and its chamber structure, with a small hollow structure on its margin that is not in *P. suratthaniensis* n. sp. In addition, the squamodiscs of *P. urceolus* have numerous rows of rodlets, that is, 14–15 rows of rodlets and a 0–1 innermost row that form complete concentric rings; but the squamodiscs of *P. suratthaniensis* n. sp. have 8–11 rows of rodlets and 1–2 innermost rows that form closed ovals [26]. *Pseudorhabdosynochus bouaini* Neifar & Euzet, 2007 from *E. costae* (Steindachner) out of Sfax, Tunisia has a sclerotized vagina similar to that of *P*. *suratthaniensis* n. sp. It is characterized by an anterior trumpet, followed by a short and heavily sclerotized primary canal progressively in the heavy primary chamber. However, the distal part of the primary chamber has two small sclerotized protuberances in *P. bouaini* (which are not in *P*. *suratthaniensis* n. sp) [27].

### *Pseudorhabdosynochus cephalopholi* n. sp.

urn:lsid:zoobank.org:act:FDF79967-ED74-4848-BBA7-DCBC12E813A8

*Type host*: *Cephalopholis sonnerati* (Valenciennes) (Perciformes, Serranidae).

*Other host*: *C. miniata* (Forsskål) (Perciformes, Serranidae)

*Type locality*: Surat Thani Province, the lower Gulf of Thailand, Southern Thailand (9°48^′^10.1^″^N 99°55^′^31.1^″^E), May 2019.

*Type-material*: Holotype, ZRC.PLA.1118; 2 paratypes, ZRC.PLA.1119-20; 2 paratypes, THNHM-Iv-19365-66; 20 paratypes, ZMKU-PM-002054-73; 2 paratypes from other host, *C. miniata*, ZMKU-PM-002074-75.

*Site in host*: Gills.

*Infection indices*: Prevalence 100% (4/4); mean intensity 62.5 individuals/fish (250/4).

*Etymology*: Species name derived from *Cephalopholis,* which is the name of the host genus (both type-host and other host). This monogenean species seems to be specific to this host genus.

#### Description (Figs. 3 and 4)

**Figure 3:**
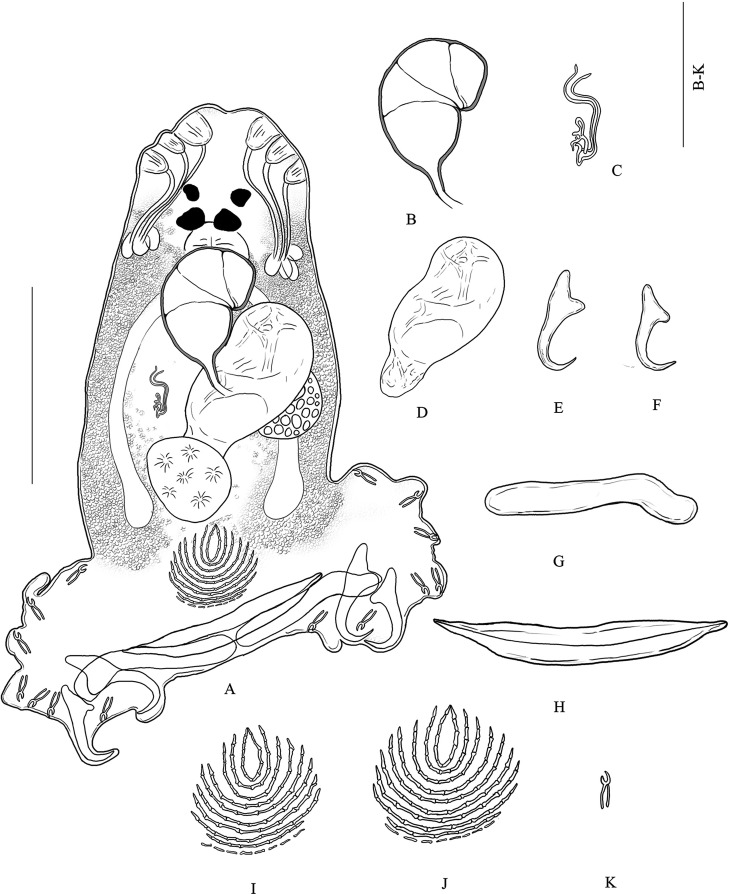
*Pseudorhabdosynochus cephalopholi* n. sp. from *Cephalopholis sonnerati* in the lower Gulf of Thailand. (A) Composite drawing (mainly from holotype), dorsal view. (B) Male quadriloculate organ, dorsal view. (C) Sclerotized vagina, dorsal view. (D) Egg. (E) Ventral hamulus. (F) Dorsal hamulus. (G) Dorsal (lateral) bar. (H) Ventral bar. (I) Dorsal squamodisc. (J) Ventral squamodisc. (K) Hooklet. Scale-bars: (A) 100 μm; (B)–(K) 50 μm.

[Based on 25 specimens]. Body (including haptor) 393 long (260–645, *n* = 25), maximum width 106 (83–126, *n* = 25). Tegument smooth. Anterior region with 3 pairs of lateral head organs and 2 pairs of eye-spots; anterior pair smaller than posterior pair. Pharynx median, spherical, 24 (18–31, *n* = 25) × 24 (19–31, *n* = 25). Esophagus absent. Intestinal bifurcation immediately follows pharynx. Haptor differentiated from rest of body, 216 wide (167–261, *n* = 25), with 2 similar squamodiscs, 2 pairs of lateral hamuli, 3 bars, and 14 marginal hooklets. Dorsal and ventral squamodiscs round-shaped, made up of rows of rodlets; rodlets similar in width in all rows except last row with thin and separate rodlets; central rows form closed ovals. Dorsal squamodisc 39 long (30–56, *n* = 11), 41 wide (32–57, *n* = 11), with 8–10 rows of rodlets, the innermost of which form closed oval. Ventral squamodisc 39 long (34–43, *n* = 11), 43 wide (37–49, *n* = 11), with 8–10 rows of rodlets, the innermost of which form closed oval. Ventral hamulus with distinct guard and expanded deep root, elongated shaft and recurved toward the tip, outer length 41 (37–46, *n* = 25), inner length 31 (29–36, *n* = 25). Dorsal hamulus with indistinct guard and expanded deep root, elongated shaft and recurved toward the tip, outer length 35 (32–38, *n* = 25), inner length 22 (19–25, *n* = 25). Dorsal (lateral) bar straight, elongated, with flattened medial extremity and cylindrical lateral extremity, 72 long (64–79, *n* = 25), 10 wide (7–14, *n* = 25). Ventral bar flat, thin, elongated, with constricted median portion and pointed ends, 104 long (90–114, *n* = 25), 10 wide (8–14, *n* = 25); visible groove that extends to both thin extremities. Male quadriloculate organ divided into 4 chambers, inner length 40 (33–46, *n* = 25), fourth chamber ends in sclerotized cone, 10 long (7–12, *n* = 25), lengthened by sclerotized tube, 11 long (6–14, *n* = 25). Testis subspherical, intercecal. Ovary pretesticular, encircles right intestinal cecum. Vitelline follicles lateral, coextensive with intestinal ceca, confluent posterior to testis region and terminate anterior to peduncle. Egg (Fig. 3D) 86 long (74–91, *n* = 4), 51 wide (38–64, *n* = 4).

**Figure 4:**
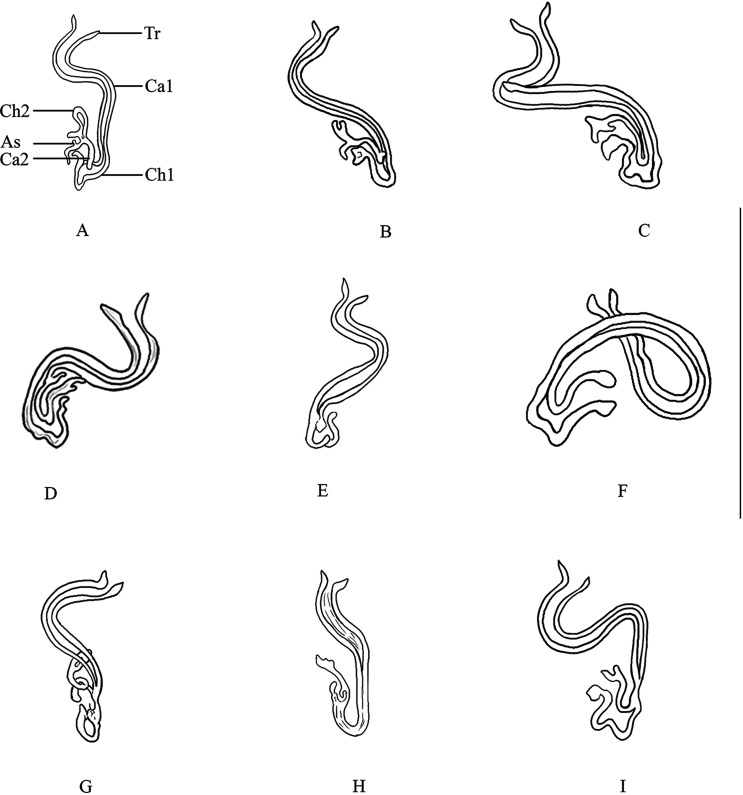
Variations (A)–(I) of the sclerotized vagina of *Pseudorhabdosynochus cephalopholi* n. sp. from *Cephalopholis sonnerati* in the lower Gulf of Thailand. Abbreviations: Tr – trumpet, Ca1 – primary canal, Ch1 – primary chamber, Ca2 – secondary canal, Ch2 – secondary chamber, As – accessory structure. Scale-bar: 50 μm.

*Sclerotized vagina* with a complex sclerotized structure, aspect changes according to specimen and orientation (Figs. 4A–4I). Sclerotized vagina comprises anterior trumpet, followed by primary canal, primary chamber, secondary canal, secondary chamber, and accessory structure; trumpet in continuity with primary canal tube, long, coiled (Fig. 4F), or curved (Figs. 4A–E and 4G–4I) at around midlength, heavily sclerotized, progressively into primary chamber or form a bend in posterior region and progressively into primary chamber (Fig. 4H); secondary chamber communicates with primary chamber through a short secondary canal, and accessory structure connected to secondary chamber. Total length of sclerotized vagina (measured from distal extremity of trumpet to base of vagina, not considering curved length along coil or curve of primary canal) 25 (19–30, *n* = 25), length variable because of variation in coil or curvature of primary canal.

#### Differential diagnosis

*Pseudorhabdosynochus cephalopholi* n. sp. is distinguished from other *Pseudorhabdosynochus* species by the structure of its sclerotized vagina, in addition to the differing lengths of some sclerotized organs and the number of rows of rodlets in each squamodisc. *Pseudorhabdosynochus minutus* from *C. sonnerati* out of the Barrier reef off Nouméa, New Caledonia has the following characteristics. The general morphology of its sclerotized vagina and its size (25 μm), body size (270–550 μm versus 260–645 μm in *P. cephalopholi* n. sp.), number of rows of rodlets in each squamodisc (10–11 rows of rodlets and a 0–1 closed oval), and host species (*C. sonnerati*) are similar to those of *P. cephalopholi* n. sp. *Pseudorhabdosynochus minutus* can be distinguished, however, by the chamber structure of its sclerotized vagina (*P. minutus* has spherical chambers, unlike the chambers in *P. cephalopholi* n. sp.). *Pseudorhabdosynochus argus* from *C. argus* out of the Barrier reef off Nouméa, New Caledonia has a sclerotized vagina similar to that of *P. cephalopholi* n. sp., characterized by an anterior trumpet followed by a long primary canal. *Pseudorhabdosynochus argus* can be distinguished, however, by the size of its sclerotized vagina (38 μm versus 25 μm in *P. cephalopholi* n. sp.); the morphology of its sclerotized vagina, with the primary canal coiled in its anterior part, just after the trumpet (see [15]), unlike in *P*. *cephalopholi* n. sp.; its body size (888 μm versus 393 μm); and the different lengths of its sclerotized organs: of its ventral hamulus, outer length of 53 μm versus 41 μm and inner length of 44 μm versus 31 μm; of its dorsal hamulus, outer length of 44 μm versus 35 μm and inner length of 27 μm versus 22 μm; of its ventral bar, 79 μm long versus 104 μm long; of its quadriloculate organ, inner length of 58 μm versus 40 μm; and of the cone of its quadriloculate organ, 22 μm long versus 10 μm long (Table 1). *Pseudorhabdosynochus* sp. Justine, 2007 from *C. boenak* (Bloch) out of Heron Island, Queensland, Australia has a sclerotized vagina similar to that of *P. cephalopholi* n. sp., characterized by an anterior trumpet followed by a long primary canal. *Pseudorhabdosynochus* sp. can be distinguished by the following: the morphology of its primary canal, the shapes of its chambers, and the number of rows of rodlets in each of its squamodiscs (7 rows of rodlets, including 0–2 closed rows versus 8–10 rows of rodlets and an innermost row closed oval in *P*. *cephalopholi* n. sp.). *Pseudorhabdosynochus* sp. generally resembles a dwarf *P. argus* [15]. *Pseudorhabdosynochus euitoe* Justine, 2007 from *E. maculatus* (Bloch) out of the Barrier reef off Nouméa, New Caledonia has a sclerotized vagina similar to that of *P*. *cephalopholi* n. sp., characterized by an anterior trumpet followed by a long, coiled primary canal. However, the trumpet is characterized by a heavily sclerotized ring at the trumpet-canal limit in the *P. euitoe* (which is absent in *P. cephalopholi* n. sp.). *Pseudorhabdosynochus fuitoe* Justine, 2007 from *E. maculatus* out of the Barrier reef off Nouméa, New Caledonia has a sclerotized vagina similar to that of *P*. *cephalopholi* n. sp., characterized by an anterior trumpet followed by a heavy, long, coiled or curved primary canal, but the shapes of their chambers differ [14]. *Pseudorhabdosynochus manifestus* Justine & Sigura, 2007 from *E. malabaricus* (Bloch & Schneider) out of the Lagoon off Nouméa, New Caledonia is differentiated by its sclerotized vagina and its cone-shaped primary canal that is coiled in its anterior part and straight in its posterior part (which is absent in *P. cephalopholi* n. sp.) [20].

**Table 1:** Measurements of *P. cephalopholi* n. sp. from the type host, *C. sonnerati*, *C. miniata*, and two previously described *Pseudorhabdosynochus* species from *Cephalopholis* spp. The measurements of *P. cephalopholi* n. sp. were taken from flattened specimens in ammonium picrate-glycerin.

Species host	*P. cephalopholi* n. sp. *C. sonnerati*	*P. cephalopholi* n. sp. *C. miniata*	*P. argus C. argus* (From Justine, 2007 [15])	*P. minutus C. sonnerati* (From Justine, 2007 [15])
Total body length	393 (260–645, *n* = 25)	408 (327–582, *n* = 8)	888 (750–1200, *n* = 16)	363 (270–550, *n* = 4)
Body width	106 (83–126, *n* = 25)	99 (80–116, *n* = 8)	260 (200–320, *n* = 16)	131 (90–170, *n* = 4)
Haptor width	216 (167–261, *n* = 25)	206 (167–240, *n* = 8)		211 (185–240, *n* = 4)
Quadriloculate organ				
Inner length	40 (33–46, *n* = 25)	40 (34–46, *n* = 8)	58 (55–63, *n* = 24)	49 (45–53, *n* = 9)
Cone length	10 (7–12, *n* = 25)	10 (9–10, *n* = 8)	22 (12–26, *n* = 24)	6 (4–7, *n* = 9)
Tube length	11 (6–14, *n* = 25)	12 (11–15, *n* = 8)	20 (15–25, *n* = 24)	10 (6–14, *n* = 9)
Sclerotized vagina length	25 (19–30, *n* = 25)	25 (21–28, *n* = 8)	38 (35–41, *n* = 24)	25 (24–30, *n* = 9)
Ventral hamulus				
Outer length	41 (37–46, *n* = 25)	41 (40–44, *n* = 8)	53 ± 2.2 (47–56, *n* = 48)	44 (41–45, *n* = 18)
Inner length	31 (29–36, *n* = 25)	31 (30–32, *n* = 8)	44 ± 1.6 (38–46, *n* = 48)	33 (30–38, *n* = 18)
Dorsal hamulus				
Outer length	35 (32–38, *n* = 25)	35 (33–38, *n* = 8)	44 ± 1.2 (42–47, *n* = 48)	37 (35–39, *n* = 18)
Inner length	22 (19–25, *n* = 25)	22 (21–24, *n* = 8)	27 ± 1.2 (24–28, *n* = 48)	25 (23–26, *n* = 18)
Ventral bar				
Length	104 (90–114, *n* = 25)	101 (97–105, *n* = 8)	79 (71–84, *n* = 24)	99 (93–104, *n* = 9)
Width	10 (8–14, *n* = 25)	9 (9–10, *n* = 8)	20 (17–23, *n* = 24)	10 (9–11, *n* = 9)
Dorsal bar				
Length	72 (64–79, *n* = 25)	73 (66–78, *n* = 8)	68 ± 3.4 (57–73, *n* = 48)	69 (63–76, *n* = 18)
Width	10 (7–14, *n* = 25)	12 (10–13, *n* = 8)	20 ± 1.6 (16–23, *n* = 48)	12 (9–15, *n* = 17)
Number of rows of rodlets	8–10 (*n* = 11), innermost row closed, oval	8–10 (*n* = 2), innermost row closed, oval	9–11 (*n* = 7), 1–2 closed ovals	10–11 (*n* = 10), 0–1 closed oval
Prevalence	100% (4/4)	56% (5/9)	100% (3/3)	100% (3/3)

### *Pseudorhabdosynochus samaesarnensis* n. sp.

urn:lsid:zoobank.org:act:EF09975F-D751-41C5-B742-154A1B5E48FE

*Type host*: *Epinephelus lanceolatus* (Bloch) (Perciformes, Serranidae).

*Other hosts*: *E. coioides* (Hamilton), *E. erythrurus* (Valenciennes), *E. coeruleopunctatus* (Bloch), *E. malabaricus* (Bloch & Schneider), sea cage-cultured hybrid grouper (TGGG) (*E. fuscoguttatus ♀ × E. lanceolatus* ♂) and *E. fuscoguttatus* (Forsskål) (Perciformes, Serranidae).

*Type-locality*: Samaesarn Island in the Gulf of Thailand, Chonburi Province, Eastern Thailand (12°34^′^30.9^″^N 100°57^′^33.7^″^E), September 2020.

*Other locality*: Libong Island in the Andaman Sea, Trang Province, Southern Thailand (7°13^′^00.3^″^N 99°21^′^36.0^″^E), April 2020; Ban Laem Hin, Phang-Nga Province in the Andaman Sea, Southern Thailand (8°10^′^55.5^″^N 98°20^′^35.3^″^E), May 2020.

*Type-material*: Holotype, ZRC.PLA.2041; 2 paratypes, ZRC.PLA.2042-43; 2 paratypes, THNHM-Iv-19367-68; 1 paratype, ZMKU-PM-002076; 2 paratypes from other host, *E. coioides*, ZMKU-PM-002077-78; 2 paratypes from other host, *E. erythrurus*, ZMKU-PM-002079-80; 2 paratypes from other host, *E. coeruleopunctatus*, ZMKU-PM-002081-82; 2 paratypes from other host, *E. malabaricus*, ZMKU-PM-002083-84; 1 paratype from other host, sea cage-cultured hybrid grouper (TGGG) (*E. fuscoguttatus* ♀ × *E. lanceolatus* ♂), ZMKU-PM-002085.

*Site in host*: Gills.

*Infection indices*: Prevalence 100% (one specimen examined and infected); 12 helminth specimens on the single grouper examined.

*Etymology*: The species name “*samaesarnensis*” was derived from “Samaesarn Island”, where the host fish *Epinephelus lanceolatus* was collected.

#### Description (Figs. 5 and 6)

[Based on 6 specimens]. Body (including haptor) 745 long (679–821, *n* = 6); maximum width 198 (160–234, *n* = 6). Tegument scaly (observed in some specimens). Anterior region with 3 pairs of lateral head organs and 2 pairs of eye-spots; anterior pair smaller than posterior pair. Pharynx median, spherical, 45 (39–47, *n* = 6) × 45 (39–47, *n* = 6). Esophagus absent. Intestinal bifurcation immediately follows pharynx. Haptor differentiated from rest of body, 220 wide (178–243, *n* = 6), with 2 similar squamodiscs, 2 pairs of lateral hamuli, 3 bars, and 14 marginal hooklets. Squamodiscs round-shaped, made up of rows of rodlets, the innermost 1–2 rows of which form closed ovals and last row of which are thinner and separated. Dorsal squamodisc 53 long (51–54, *n* = 4), 47 wide (44–50, *n* = 4), with 9–12 rows of rodlets, the innermost 1–2 rows of which form closed ovals. Ventral squamodisc 52 long (49–56, *n* = 4), 45 wide (41–52, *n* = 4), with 9–10 rows of rodlets, the innermost 1–2 rows of which form closed ovals. Ventral hamulus with distinct guard and expanded deep root, elongated shaft slightly arched and recurved toward the tip, outer length 48 (45–51, *n* = 6), inner length 37 (35–39, *n* = 6). Dorsal hamulus with indistinct guard and expanded deep root, elongated shaft slightly arched and recurved toward the tip, outer length 41 (40–41, *n* = 6), inner length 25 (24–27, *n* = 6). Dorsal (lateral) bar straight, with flattened medial extremity, 61 long (57–67, *n* = 6), 17 wide (16–19, *n* = 6). Ventral bar elongated, with constricted median portion and tapered ends, 94 long (92–96, *n* = 6), 15 wide (14–16, *n* = 6); visible groove that extends to both thin extremities. Male quadriloculate organ with fourth (posterior) chamber slightly more sclerotized than 3 anterior chambers, inner length 56 (51–60, *n* = 6), fourth chamber ends in sclerotized cone, 12 long (10–13, *n* = 6), prolonged by sclerotized tube, 15 long (13–17, *n* = 6), end of tube prolonged by filament of variable length. Test is subspherical, intercecal. Ovary pretesticular, encircles right intestinal cecum. Vitelline follicles lateral, coextensive with intestinal ceca and terminate anterior to the peduncle. Egg not seen.

**Figure 5:**
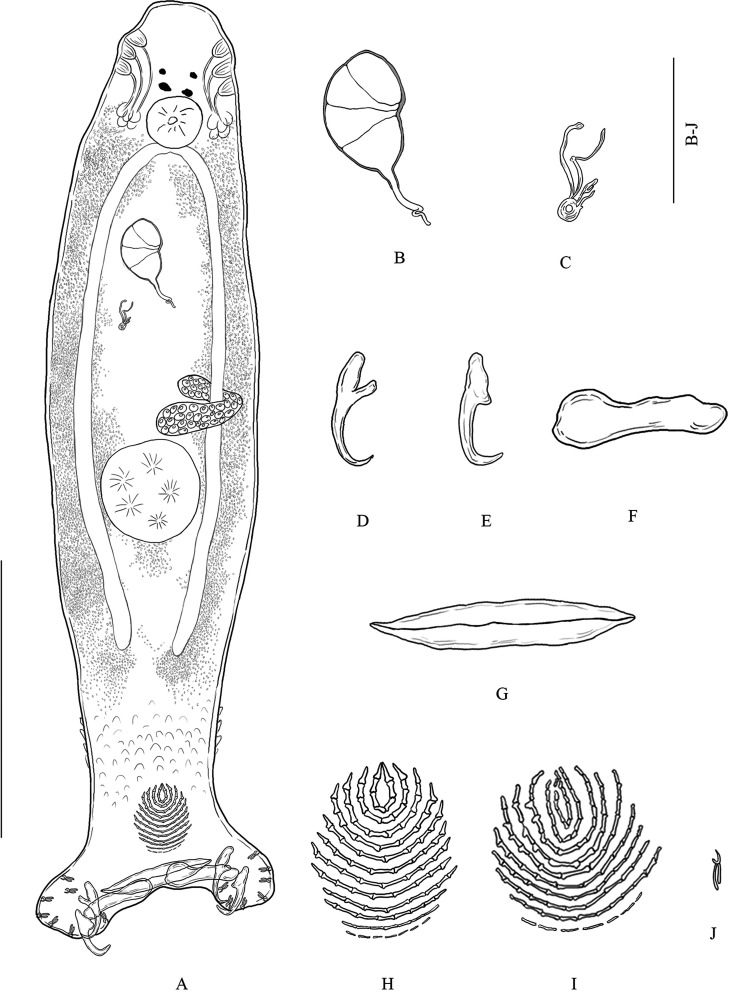
*Pseudorhabdosynochus samaesarnensis* n. sp. from *Epinephelus lanceolatus* from Samaesarn Island in the Gulf of Thailand. (A) Composite drawing (mainly from holotype), dorsal view. (B) Male quadriloculate organ, dorsal view. (C) Sclerotized vagina, dorsal view. (D) Ventral hamulus. (E) Dorsal hamulus. (F) Dorsal (lateral) bar. (G) Ventral bar. (H) Dorsal squamodisc. (I) Ventral squamodisc. (J) Hooklet. Scale-bars: (A) 200 μm. (B)–(J): 50 μm.

**Figure 6:**
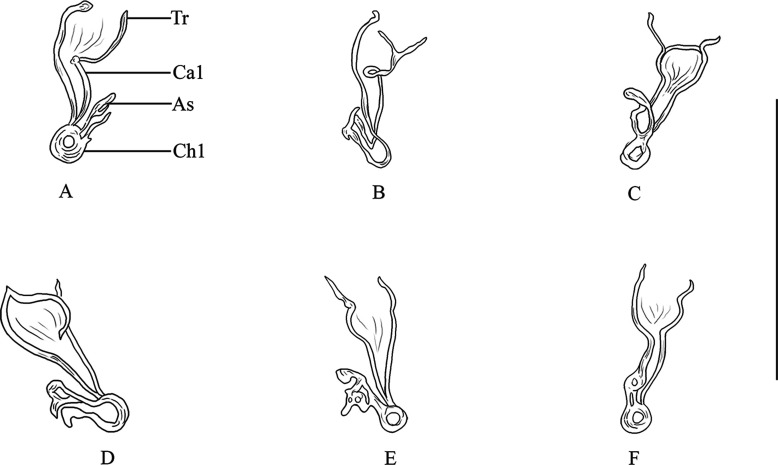
Variations (A)–(F) of the sclerotized vagina of *Pseudorhabdosynochus samaesarnensis* n. sp. from *Epinephelus lanceolatus* and *E. coioides* in the Gulf of Thailand. Abbreviations: Tr – trumpet, Ca1 – primary canal, Ch1 – primary chamber. As, accessory structure. Scale-bar: 50 μm.

*Sclerotized vagina* with a complex sclerotized structure, aspect changes according to specimen and orientation (Figs. 6A–6F). Sclerotized vagina comprises anterior cup-shaped trumpet, followed by primary canal, primary chamber and accessory structure; thick-walled anterior cup-shaped trumpet in continuity with short straight or curved primary canal (Fig. 6A), thick-walled sclerotization and widens into a thick-walled primary chamber; accessory structure connects to the primary chamber. Total length of sclerotized vagina (measured from distal extremity of trumpet to base of vagina, not considering curve along primary canal) 27 (25–29, *n* = 6), length variable because of variation in curvature of primary canal.

#### Differential diagnosis

*Pseudorhabdosynochus samaesarnensis* n. sp. is distinguished from other *Pseudorhabdosynochus* species by the structure of its sclerotized vagina. *Pseudorhabdosynochus samaesarnensis* n. sp. has a vaginal structure similar to that of *P. nhatrangensis* from *E. coioides* (Hamilton) and *E. bleekeri* (Vaillant) off Vietnam. The structure of their sclerotized vagina was found to be very similar, but the number of rows of rodlets found in *P. nhatrangensis* was always 10 [7], while in *P. samaesarnensis* n. sp., the rows were found to vary in number from 9 to 13. In addition, the specimens of *P. samaesarnensis* n. sp. from *E. lanceolatus* were larger than those of *P. nhatrangensis* from *E. coioides* and *E. bleekeri* (Table 2). However, *P. nhatrangensis* Dang, Bristow, Schander & Berland, 2013 is not a valid species because the article was not compliant with the new Article 8.5.3 of the International Code of Zoological Nomenclature (ICZN) [3].

**Table 2:** Measurements of specimens of *P. samaesarnensis* n. sp. from the type host, *E. lanceolatus* from the Gulf of Thailand, compared with *P. nhatrangensis* from *E. coioides* and *E. bleekeri* from Nha Trang Bay, Vietnam.

Species host	*P. samaesarnensis* n. sp. *E. lanceolatus*	*P. nhatrangensis E. coioides* and *E. bleekeri* (details from Dang et al., 2013 [7])
Total body length	745 (679–821, *n* = 6)	458 (370–570, *n* = 10)
Body width	198 (160–234, *n* = 6)	155 (120–185, *n* = 10)
Haptor width	220 (178–243, *n* = 6)	157 (130–188, *n* = 7)
Quadriloculate organ		
Inner length	56 (51–60, *n* = 6)	58 (52–65, *n* = 10)
Cone length	12 (10–13, *n* = 6)	14 (12–15, *n* = 10)
Tube length	15 (13–17, *n* = 6)	21 (18–25, *n* = 10)
Sclerotized vagina length	27 (25–29, *n* = 6)	29 (25–33, *n* = 10)
Ventral hamulus		
Outer length	48 (45–51, *n* = 6)	48 (40–58, *n* = 10)
Inner length	37 (35–39, *n* = 6)	38 (33–40, *n* = 10)
Dorsal hamulus		
Outer length	41 (40–41, *n* = 6)	38 (33–43, *n* = 10)
Inner length	25 (24–27, *n* = 6)	24 (23–25, *n* = 10)
Ventral bar		
Length	94 (92–96, *n* = 6)	95 (90–103, *n* = 10)
Width	15 (14–16, *n* = 6)	11 (9–14, *n* = 10)
Dorsal bar		
Length	61 (57–67, *n* = 6)	61 (55–68, *n* = 10)
Width	17 (16–19, *n* = 6)	12 (11–14, *n* = 10)
Number of rows of rodlets	9–12 (*n* = 4) including 1–2 closed ovals	Always 10 (*n* = 6) and 1 closed circle
Prevalence	100% (one specimen examined and infected)	27% (12/45) on *E. coioides* and 10% (4/40) on *E. bleekeri*

Note that *P. nhatrangensis* is not a valid species.

## Discussion

Species of *Pseudorhabdosynochus* parasitize marine fish, especially groupers (Serranidae, Epinephelinae) [3, 5, 21, 24, 33], and have hitherto been considered strictly host-specific or “specialists,” that is, found in a single species [12–16, 20, 22, 33]. However, other *Pseudorhabdosynochus* species occur on several host species, such as *P. cyanopodus* Sigura & Justine, 2008 on *E. cyanopodus* (Richardson) and *E. chlorostigma* (Valenciennes) [19, 33, 36]; *P. amplidiscatum* (Bravo-Hollis, 1954) Kritsky & Beverley Burton, 1986 on *E. analogus* (Gill) and *E. labriformis* (Jenyns); *P. guerreroensis* Mendoza-Franco, Violante-González & Herrera, 2011 on *Alphestes immaculatus* (Breder), *A. multiguttatus* (Günther) and *E. analogus* [26]; *P. huitoe* Justine, 2007 on *E. maculatus* and *E. cyanopodus* [14, 33]; *P. firmicoleatus* Kritsky, Bakenhaster & Adams, 2015 on *Hyporthodus flavolimbatus* (Poey) and *H. niveatus* (Valenciennes); and *P. sulamericanus* Santos, Buchmann & Gibson, 2000 on *H. niveatus*, *H. nigritus* (Holbrook), and *H. haifensis* (Ben-Tuvia) [4, 24, 34]. The sclerotized vaginae are characteristic of individual species and are important for species identification [5, 12–14, 17, 21, 23, 26, 31–33].

Three new species of *Pseudorhabdosynochus* are described. *Pseudorhabdosynochus suratthaniensis* n. sp. was found on a single host species, *C. argus*, while *P. cephalopholi* n. sp. and *P. samaesarnensis* n. sp. were found on two host species and seven host species, respectively. *Pseudorhabdosynochus cephalopholi* n. sp. was found on *C. sonnerati* and *C. miniata*, and its morphometric data from both *C. sonnerati* and *C. miniata* are similar in size (Table 1). In addition, the structures of the sclerotized vaginae are no different (*P. cephalopholi* n. sp., which is rare in *C. miniata*). *Pseudorhabdosynochus samaesarnensis* n. sp. was found on seven host species: *E. lanceolatus*, *E. coioides*, *E. erythrurus*, *E. coeruleopunctatus*, *E. malabaricus*, sea cage-cultured hybrid grouper (TGGG) (*E. fuscoguttatus ♀ × E. lanceolatus* ♂), and *E. fuscoguttatus*, and the morphometric data of *P. samaesarnensis* n. sp. from the six hosts are similar in size (Table 3). In addition, the structures of the sclerotized vaginae are no different. This table does not include the details of *P. samaesarnensis* n. sp. from *E. fuscoguttatus* because its specimens are incomplete and are found in low numbers (prevalence: about 9.1%). *Pseudorhabdosynochus samaesarnensis* n. sp. has a structure of the sclerotized vagina different from other species previously found in host fish – *E. coioides* [1, 9, 37–39], *E. erythrurus* [30], *E. coeruleopunctatus* [35], *E. malabaricus* [20], and sea cage-cultured hybrid grouper (TGGG) (*E. fuscoguttatus* ♀ × *E. lanceolatus* ♂) [8]. We hypothesized that *P. cephalopholi* n. sp. and *P. samaesarnensis* n. sp. have low specificity to their hosts and that their infestations of more than one congeneric host species help to perpetuate this parasitic species [36].

**Table 3:** Measurements of *P. samaesarnensis* n. sp. from the type host, *E. lanceolatus* and its other hosts, *E. coioides*, *E. erythrurus*, *E. coeruleopunctatus*, *E. malabaricus*, and sea cage-cultured hybrid grouper (TGGG) (*E. fuscoguttatus* ♀ × *E. lanceolatus* ♂).

Host	*E. lanceolatus*	*E. coioides*	*E. erythrurus*	*E. coeruleopunctatus*	*E. malabaricus*	Sea cage-cultured hybrid grouper (TGGG)
Total body length	745 (679–821, *n* = 6)	657 (436–826, *n* = 10)	666 (598–799, *n* = 10)	644 (437–774, *n* = 10)	618 (368–777, *n* = 10)	732 (624–857, *n* = 10)
Body width	198 (160–234, *n* = 6)	143 (92–179, *n* = 10)	134 (88–180, *n* = 10)	135 (108–162, *n* = 10)	132 (94–161, *n* = 10)	130 (97–173, *n* = 10)
Haptor width	220 (178–243, *n* = 6)	186 (150–221, *n* = 10)	182 (147–229, *n* = 10)	189 (156–219, *n* = 10)	182 (148–228, *n* = 10)	198 (161–222, *n* = 10)
Quadriloculate organ						
Inner length	56 (51–60, *n* = 6)	53 (46–57, *n* = 10)	52 (46–56, *n* = 10)	51 (43–57, *n* = 10)	51 (44–59, *n* = 10)	52 (44–58, *n* = 10)
Cone length	12 (10–13, *n* = 6)	12 (10–13, *n* = 10)	12 (10–14, *n* = 10)	12 (10–14, *n* = 10)	12 (10–13, *n* = 10)	11 (10–13, *n* = 10)
Tube length	15 (13–17, *n* = 6)	13 (11–15, *n* = 10)	13 (12–15, *n* = 10)	13 (11–16, *n* = 10)	14 (11–15, *n* = 10)	14 (12–15, *n* = 10)
Sclerotized vagina length	27 (25–29, *n* = 6)	25 (23–27, *n* = 10)	27 (25–29, *n* = 10)	27 (24–29, *n* = 10)	25 (23–28, *n* = 10)	27 (24–31, *n* = 10)
Ventral hamulus						
Outer length	48 (45–51, *n* = 6)	43 (40–45, *n* = 10)	43 (41–45, *n* = 10)	44 (41–46, *n* = 10)	45 (41–48, *n* = 10)	45 (43–48, *n* = 10)
Inner length	37 (35–39, *n* = 6)	34 (33–36, *n* = 10)	34 (32–36, *n* = 10)	34 (33–36, *n* = 10)	36 (34–37, *n* = 10)	35 (34–37, *n* = 10)
Dorsal hamulus						
Outer length	41 (40–41, *n* = 6)	38 (37–40, *n* = 10)	39 (37–40, *n* = 10)	39 (38–40, *n* = 10)	39 (36–41, *n* = 10)	39 (38–41, *n* = 10)
Inner length	25 (24–27, *n* = 6)	25 (23–26, *n* = 10)	25 (24–26, *n* = 10)	25 (24–27, *n* = 10)	25 (24–27, *n* = 10)	25 (23–27, *n* = 10)
Ventral bar						
Length	94 (92–96, *n* = 6)	90 (85–98, *n* = 10)	92 (84–100, *n* = 10)	92 (83–96, *n* = 10)	87 (82–92, *n* = 10)	87 (83–95, *n* = 10)
Width	15 (14–16, *n* = 6)	14 (12–16, *n* = 10)	14 (12–15, *n* = 10)	15 (13–17, *n* = 10)	15 (10–17, *n* = 10)	15 (12–17, *n* = 10)
Dorsal bar						
Length	61 (57–67, *n* = 6)	58 (56–60, *n* = 10)	55 (50–59, *n* = 10)	56 (53–59, *n* = 10)	58 (53–63, *n* = 10)	59 (55–64, *n* = 10)
Width	17 (16–19, *n* = 6)	17 (14–20, *n* = 10)	15 (12–17, *n* = 10)	17 (14–20, *n* = 10)	18 (13–21, *n* = 10)	18 (13–21, *n* = 10)
Number of rows of rodlets	9–12 (*n* = 4) including 1–2 closed ovals	10–13 (*n* = 4) including 1–2 closed ovals	9–12 (*n* = 5) including 1–2 closed ovals	10–11 (*n* = 2) including 1–2 closed ovals	10–12 (*n* = 7) including 1–2 closed ovals	10–12 (*n* = 5) including 1–2 closed ovals
Prevalence	100% (one specimen examined and infected)	100% (4/4)	100% (5/5)	100% (4/4)	100% (4/4)	100% (3/3)

## References

[1] Bu SSH, Leong TS, Wong SY, Woo YSN, Foo RWT. 1999. Three diplectanid monogeneans from marine finfish (*Epinephelus* spp.) in the Far East. Journal of Helminthology, 73, 301–312.

[2] Ch’ng CL, Senoo S. 2008. Egg and larval development of a new hybrid grouper, tiger grouper *Epinephelus fuscoguttatus* × giant grouper *E. lanceolatus*. Aquaculture Science, 56(4), 505–512.

[3] Chaabane A, Neifar L, Justine J-L. 2015. *Pseudorhabdosynochus regius* n. sp. (Monogenea, Diplectanidae) from the mottled grouper *Mycteroperca rubra* (Teleostei) in the Mediterranean Sea and Eastern Atlantic. Parasite, 22, 9. 25674913 10.1051/parasite/2015005 PMC4325681

[4] Chaabane A, Justine J-L, Gey D, Bakenhaster MD, Neifar L. 2016. *Pseudorhabdosynochus sulamericanus* (Monogenea, Diplectanidae), a parasite of deep-sea groupers (Serranidae) occurs transatlantically on three congeneric hosts (*Hyporthodus* spp.), one from the Mediterranean Sea and two from the western Atlantic. PeerJ, 4, e2233. 27602259 10.7717/peerj.2233 PMC4991870

[5] Chaabane A, Neifar L, Gey D, Justine J-L. 2016. Species of *Pseudorhabdosynochus* (Monogenea, Diplectanidae) from groupers (*Mycteroperca* spp., Epinephelidae) in the Mediterranean and Eastern Atlantic Ocean, with special reference to the ‘Beverleyburtonae group’ and description of two new species. PLoS One, 11, e0159886. 27532108 10.1371/journal.pone.0159886 PMC4988817

[6] Ching FF, Othman N, Anuar A, Shapawi R, Senoo S. 2018. Natural spawning, embryonic and larval development of F2 hybrid grouper, tiger grouper *Epinephelus fuscoguttatus* × giant grouper *E. lanceolatus*. International Aquatic Research, 10, 391–402.

[7] Dang BT, Bristow GA, Schander C, Berland B. 2013. Three new species of *Pseudorhabdosynochus* (Monogenea: Diplectanidae) from Vietnamese grouper (*Epinephelus* spp.) (Perciformes: Serranidae). International Journal of Aquatic Science, 4, 44–58.

[8] Dewi NTB, Aryadi IF, Arrizal AFT, Mardika DR, Syahputra PA, Subekti S, Kismiyati Sari PDW. 2018. Monogenean parasites on cantang grouper (*Epinephelus fuscoguttatus*-*lanceolatus*) wilture in floating net cage for mariculture center Lombok, West Nusa Tenggara, Indonesia. IOP Conference Series: Earth and Environmental Science, 137, 012053.

[9] Erazo-Pagador G, Cruz-Lacierda ER. 2010. The morphology and life cycle of the gill monogenean (*Pseudorhabdosynochus lantauensis*) on orange-spotted grouper (*Epinephelus coioides*) cultured in the Philippines. Bulletin of the European Association of Fish Pathologists, 30(2), 55–64.

[10] Gibson D, Editor. 2021. Pseudorhabdosynochus Yamaguti, 1958, WoRMS (World Register of Marine Species). Available on line at: http://www.marinespecies.org/aphia.php?p=taxdetails&id=468117. (Last accessed 10 December 2021).

[11] Heemstra PC, Randall JE. 1993. FAO Species Catalogue. Vol. 16. Groupers of the world (Family Serranidae, Subfamily Epinephelinae). An annotated and illustrated catalogue of the grouper, rockcod, hind, coral grouper and lyretail species known to date. . Rome, FAO, FAO Fisheries Synopsis, 125(16), 382. 31 colour plates

[12] Justine J-L. 2005. *Pseudorhabdosynochus hirundineus* n. sp. (Monogenea: Diplectanidae) from *Variola louti* (Perciformes: Serranidae) off New Caledonia. Systematic Parasitology, 62, 39–45. 16132869 10.1007/s11230-005-5481-z

[13] Justine J-L. 2005. Species of *Pseudorhabdosynochus* Yamaguti, 1958 (Monogenea: Diplectanidae) from *Epinephelus fasciatus* and *E*. *merra* (Perciformes: Serranidae) off New Caledonia and other parts of the Indo-Pacific Ocean, with a comparison of measurements of specimens prepared using different methods, and a description of *P*. *caledonicus* n. sp. Systematic Parasitology, 62, 1–37. 16132868 10.1007/s11230-005-5480-0

[14] Justine J-L. 2007. Parasite biodiversity in a coral reef fish: Twelve species of monogeneans on the gills of the grouper *Epinephelus maculatus* (Perciformes: Serranidae) off New Caledonia, with a description of eight new species of *Pseudorhabdosynochus* (Monogenea: Diplectanidae). Systematic Parasitology, 66, 81–129. 16972153 10.1007/s11230-006-9057-3

[15] Justine J-L. 2007. *Pseudorhabdosynochus argus* n. sp. (Monogenea: Diplectanidae) from *Cephalopholis argus*, *P*. *minutus* n. sp. and *Diplectanum nanus* n. sp. from *C*. *sonnerati* and other monogeneans from *Cephalopholis* spp. (Perciformes: Serranidae) off Australia and New Caledonia. Systematic Parasitology, 68, 195–215. 17896188 10.1007/s11230-007-9096-4

[16] Justine J-L. 2008. Two new species of *Pseudorhabdosynochus* Yamaguti, 1958 (Monogenea: Diplectanidae) from the deep-sea grouper *Epinephelus morrhua* (Val.) (Perciformes: Serranidae) off New Caledonia. Systematic Parasitology, 71, 145–158. 18716902 10.1007/s11230-008-9156-4

[17] Justine J-L. 2009. A redescription of *Pseudorhabdosynochus epinepheli* (Yamaguti, 1938), the type-species of *Pseudorhabdosynochus* Yamaguti, 1958 (Monogenea: Diplectanidae), and the description of *P*. *satyui* n. sp. from *Epinephelus akaara* off Japan. Systematic Parasitology, 72, 27–55. 19048406 10.1007/s11230-008-9171-5

[18] Justine J-L. 2010. Parasites of coral reef fish: how much do we know? With a bibliography of fish parasites in New Caledonia. Belgian Journal of Zoology, 140(Suppl.), 155–190.

[19] Justine J-L, Henry É. 2010. Monogeneans from *Epinephelus chlorostigma* (Val.) (Perciformes: Serranidae) off New Caledonia, with the description of three new species of diplectanids. Systematic Parasitology, 77, 81–105. 20852982 10.1007/s11230-010-9263-x

[20] Justine J-L, Sigura A. 2007. Monogeneans of the malabar grouper *Epinephelus malabaricus* (Perciformes, Serranidae) off New Caledonia, with a description of six new species of *Pseudorhabdosynochus* (Monogenea: Diplectanidae). Zootaxa, 1543, 1–44.

[21] Justine J-L, Vignon M. 2009. Monogeneans of the grouper *Epinephelus tauvina* (Perciformes, Serranidae) off Moorea, French Polynesia, with a description of *Pseudorhabdosynochus pai* n. sp. (Monogenea: Diplectanidae). Systematic Parasitology, 72, 113–125. 19115085 10.1007/s11230-008-9159-1

[22] Justine J-L, Beveridge I, Boxshall GA, Bray RA, Moravec F, Trilles J-P, Whittington ID. 2010. An annotated list of parasites (Isopoda, Copepoda, Monogenea, Digenea, Cestoda and Nematoda) collected in groupers (Serranidae, Epinephelinae) in New Caledonia emphasizes parasite biodiversity in coral reef fish. Folia Parasitologica, 57, 237–262. 21344838 10.14411/fp.2010.032

[23] Knoff M, Cohen SC, Cárdenas MQ, Cárdenas-Callirgos JM, Gomes DC. 2015. A new species of diplectanid (Monogenoidea) from *Paranthias colonus* (Perciformes, Serranidae) off Peru. Parasite, 22, 11. 25754099 10.1051/parasite/2015011 PMC4353888

[24] Kritsky DC, Bakenhaster MD, Adams DH. 2015. *Pseudorhabdosynochus* species (Monogenoidea, Diplectanidae) parasitizing groupers (Serranidae, Epinephelinae, Epinephelini) in the western Atlantic Ocean and adjacent waters, with descriptions of 13 new species. Parasite, 22, 24. 26272242 10.1051/parasite/2015024 PMC4536336

[25] Malmberg G. 1957. Om förekomsten av *Gyrodactylus* på svenska fiskar. Skrifter Utgivna av Södra Sveriges Fiskeriförening. Årsskrift, 1956, 19–76.

[26] Mendoza-Franco EF, Violante-González J, Herrera AAR. 2011. Six new and one previously described species of *Pseudorhabdosynochus* (Monogenoidea, Diplectanidae) infecting the gills of groupers (Perciformes, Serranidae) from the Pacific coasts of Mexico and Panama. Journal of Parasitology, 97, 20–35. 21348602 10.1645/GE-2716.1

[27] Neifar L, Euzet L. 2007. Five new species of *Pseudorhabdosynochus* (Monogenea: Diplectanidae) from the gills of *Epinephelus costae* (Teleostei: Serranidae). Folia Parasitologica, 54, 117–128. 17886741 10.14411/fp.2007.017

[28] Nelson JS, Grande TC, Wilson MVH. 2016. Fishes of the world, 5th edition. John Wiley & Sons: Hoboken, New Jersey.

[29] Satapoomin U. 2011. The fishes of Southwestern Thailand, The Andaman Sea – A review of research and a provisional checklist of species. Phuket Marine Biological Center Research Bulletin, 70, 29–77.

[30] Saengpheng C, Purivirojkul W. 2020. *Pseudorhabdosynochus kasetsartensis* n. sp. (Monogenea: Diplectanidae) from the cloudy grouper *Epinephelus erythrurus* (Valenciennes) (Perciformes: Serranidae) in the lower Gulf of Thailand. Systematic Parasitology, 97, 99–106. 31912419 10.1007/s11230-019-09899-z

[31] Schoelinck C, Justine J-L. 2011. Four species of *Pseudorhabdosynochus* (Monogenea: Diplectanidae) from the camouflage grouper *Epinephelus polyphekadion* (Perciformes: Serranidae) off New Caledonia. Systematic Parasitology, 79, 41–61. 21487947 10.1007/s11230-010-9289-0

[32] Schoelinck C, Justine J-L. 2011. *Pseudorhabdosynochus quadratus* n. sp. (Monogenea: Diplectanidae) from the white˗streaked grouper *Epinephelus ongus* (Bloch) (Perciformes: Serranidae) off New Caledonia. Systematic Parasitology, 79, 77–80. 21487950 10.1007/s11230-011-9295-x

[33] Sigura A, Justine J-L. 2008. Monogeneans of the speckled blue grouper, *Epinephelus cyanopodus* (Perciformes, Serranidae), from off New Caledonia, with a description of four new species of *Pseudorhabdosynochus* and one new species of *Laticola* (Monogenea: Diplectanidae), and evidence of monogenean faunal changes according to the size of fish. Zootaxa, 1695, 1–44.

[34] Santos CP, Buchmann K, Gibson DI. 2000. *Pseudorhabdosynochus* spp. (Monogenea: Diplectanidae) from the gills of *Epinephelus* spp. in Brazilian waters. Systematic Parasitology, 45, 145–153. 10743859 10.1023/a:1006232029426

[35] Sigura A, Chauvet C, Justine J-L. 2007. *Pseudorhabdosynochus bacchus* sp. nov. (Monogenea, Diplectanidae) from *Epinephelus coeruleopunctatus* (Perciformes, Serranidae) off New Caledonia. Acta Parasitologica, 52(3), 196–200.

[36] Schoelinck C, Cruaud C, Justine J-L. 2012. Are all species of *Pseudorhabdosynochus* strictly host specific? – A molecular study. Parasitology International, 61, 356–359. 22326703 10.1016/j.parint.2012.01.009

[37] Wu XY, Chilton NB, Zhu XQ, Xie MQ, Li AX. 2005. Molecular and morphological evidence indicates that *Pseudorhabdosynochus lantauensis* (Monogenea: Diplectanidae) represents two species. Parasitology, 130, 669–677. 15977904 10.1017/s0031182004007152

[38] Yang TB, Gibson DI, Zeng BJ. 2005. *Pseudorhabdosynochus summanoides* n. sp. (Monogenea: Diplectanidae) from *Epinephelus coioides* in Dapeng Bay, South China Sea, with observations on several similar species of *Pseudorhabdosynochus* Yamaguti, 1958. Systematic Parasitology, 62, 221–239. 16315082 10.1007/s11230-005-5497-4

[39] Yang T, Zeng B, Gibson DI. 2005. Description of *Pseudorhabdosynochus shenzhenensis* n. sp. (Monogenea: Diplectanidae) and redescription of P. serrani Yamaguti, 1953 from Epinephelus coioides off Dapeng Bay, Shenzhen, China. Journal of Parasitology, 91(4), 808–813. 17089747 10.1645/GE-518R.1

